# A20 mRNA therapeutics ameliorate systemic sclerosis by suppressing TRAF-6/NF-KB signaling and DREAM expression and exerting antifibrotic effects

**DOI:** 10.3389/fimmu.2025.1665998

**Published:** 2025-10-16

**Authors:** A Ram Lee, Su Been Jeon, Hye Won Kwak, Suh Won Yang, Yoo Jin Bang, Seon-Yeong Lee, So Won Lee, Jin Hyung Park, Se Gyeong Han, Jeong Won Choi, Hyo-Jung Park, Mi-La Cho, Jae-Hwan Nam

**Affiliations:** ^1^ Lab of Translational ImmunoMedicine, Catholic Research Institute of Medical Science, College of Medicine, College of Medicine, The Catholic University of Korea, Seoul, Republic of Korea; ^2^ Department of Pathology, College of Medicine, The Catholic University of Korea, Seoul, Republic of Korea; ^3^ Department of Medical Sciences, Graduate School of The Catholic University of Korea, Seoul, Republic of Korea; ^4^ SML Biopharm, Gwanmyeong-si, Gyeonggi-do, Republic of Korea; ^5^ Department of Medical and Biological Sciences, The Catholic University of Korea, Bucheon, Republic of Korea; ^6^ BK21 Four, Department of Biotechnology, The Catholic University of Korea, Bucheon, Gyeonggi-do, Republic of Korea

**Keywords:** systemic sclerosis, A20 (TNFAIP3), dream, fibrosis, immune cells

## Abstract

**Introduction:**

Systemic sclerosis (SSc) is a chronic autoimmune disorder characterized by progressive fibrosis, vascular abnormalities, and immune dysregulation. Decreased expression of A20 (*TNFAIP3*), a key negative regulator of inflammation, has been shown to aggravate SSc pathogenesis by enhancing fibroblast activation and promoting collagen production. This study explores the therapeutic efficacy of A20 mRNA-lipid nanoparticle (LNP) delivery in restoring A20 expression and mitigating fibrosis through modulation of the TRAF6/NF-κB and Downstream Regulatory Element Antagonist Modulator (DREAM) -SMAD2 signaling pathways.

**Methods:**

Human dermal fibroblasts were transfected with A20 mRNA -LNP and subsequently stimulated with transforming growth factor-beta (TGF-β). Protein expression levels and fibrotic markers were analyzed by Western blotting and quantitative PCR. In vivo, a bleomycin-induced mouse model of SSc received weekly intramuscular injections of A20 mRNA -LNP. The extent of fibrosis was assessed through histological analysis and immunohistochemistry.

**Results:**

Transfection with A20 mRNA-LNP significantly suppressed TRAF6/NF-κB signaling and reduced fibrotic marker expression *in vitro*. In the SSc mouse model, A20 mRNA-LNP treatment markedly attenuated skin and lung fibrosis and decreased collagen deposition. Importantly, A20 overexpression led to downregulation of DREAM *in vivo* and inhibition of SMAD2 phosphorylation *in vitro*, indicating crosstalk between inflammatory and fibrotic pathways.

**Discussion:**

A20 mRNA therapy effectively alleviates fibrosis by restoring A20 expression and inhibiting TRAF6/NF-κB signaling, while also downregulating DREAM, a previously unrecognized target. This dual-pathway regulation underscores the role of A20 and DREAM as central modulators of fibrotic progression. These findings highlight the potential of A20 mRNA-LNP as a novel therapeutic strategy for SSc, offering a multifaceted approach that may surpass current treatment options by simultaneously targeting interconnected pathogenic pathways.

## Introduction

Systemic sclerosis (SSc), also known as scleroderma, is a chronic autoimmune disease marked by widespread fibrosis of the skin and internal organs, accompanied by vascular abnormalities and immune dysregulation. The disease is driven by endothelial injury, aberrant fibroblast activation, and excessive deposition of extracellular matrix proteins, ultimately leading to progressive tissue stiffening (1, 2).

A20—a ubiquitin-editing enzyme encoded by TNFAIP3—regulates fibroblast activation, collagen and α-smooth muscle actin (α-SMA) production, and NF-κB signaling (3, 4). Mechanistically, A20 inhibits NF-κB by deubiquitinating TRAF6 and disrupting its interaction with E2 ubiquitin-conjugating enzymes such as Ubc13, thereby modulating both inflammatory and fibrotic responses (5). Indeed, several genetic variants within the TNFAIP3 locus have been implicated in systemic sclerosis (SSc). The intronic SNP rs5029939 has been identified as a risk allele for SSc (6). In addition, the intronic SNP rs117480515 was shown to correlate with decreased TNFAIP3 mRNA levels (7).

The downregulation of A20 in SSc is mediated by intracellular signaling pathways. Transforming growth factor-beta (TGF-β), a central profibrotic cytokine, represses A20 expression via Smad-dependent signaling, thereby promoting fibroblast activation and collagen synthesis. Moreover, the transcriptional repressor Downstream Regulatory Element Antagonist Modulator (DREAM) directly binds to the A20 promoter, further suppressing its expression (2, 8). Evidence from mouse models supports the role of A20 as an endogenous inhibitor of fibrosis: fibroblast-specific deletion of A20 exacerbates bleomycin-induced skin and lung fibrosis, whereas DREAM-deficient mice, which display elevated A20 expression, are protected from fibrotic progression, highlighting the importance of the A20–DREAM regulatory axis (9).

Current strategies to enhance A20 expression, such as AdipoRon or lipopolysaccharide stimulation, have important limitations because they rely on endogenous transcriptional activation and therefore may not be effective in settings where A20 induction is impaired (10). In contrast, mRNA-based delivery of A20 enables the direct restoration of functional protein, independent of host transcriptional activity, and thus represents a more robust approach with the potential for sustained therapeutic benefit.

We hypothesized that mRNA-based delivery of A20 could restore its regulatory function and suppress fibrosis in SSc. To investigate this, we developed an A20 mRNA-based therapeutic approach targeting the A20–DREAM regulatory axis to modulate fibroblast activation and fibrotic responses.

## Materials and methods

### A20 gene synthesis and *in vitro* transcription

**Figure 1:**
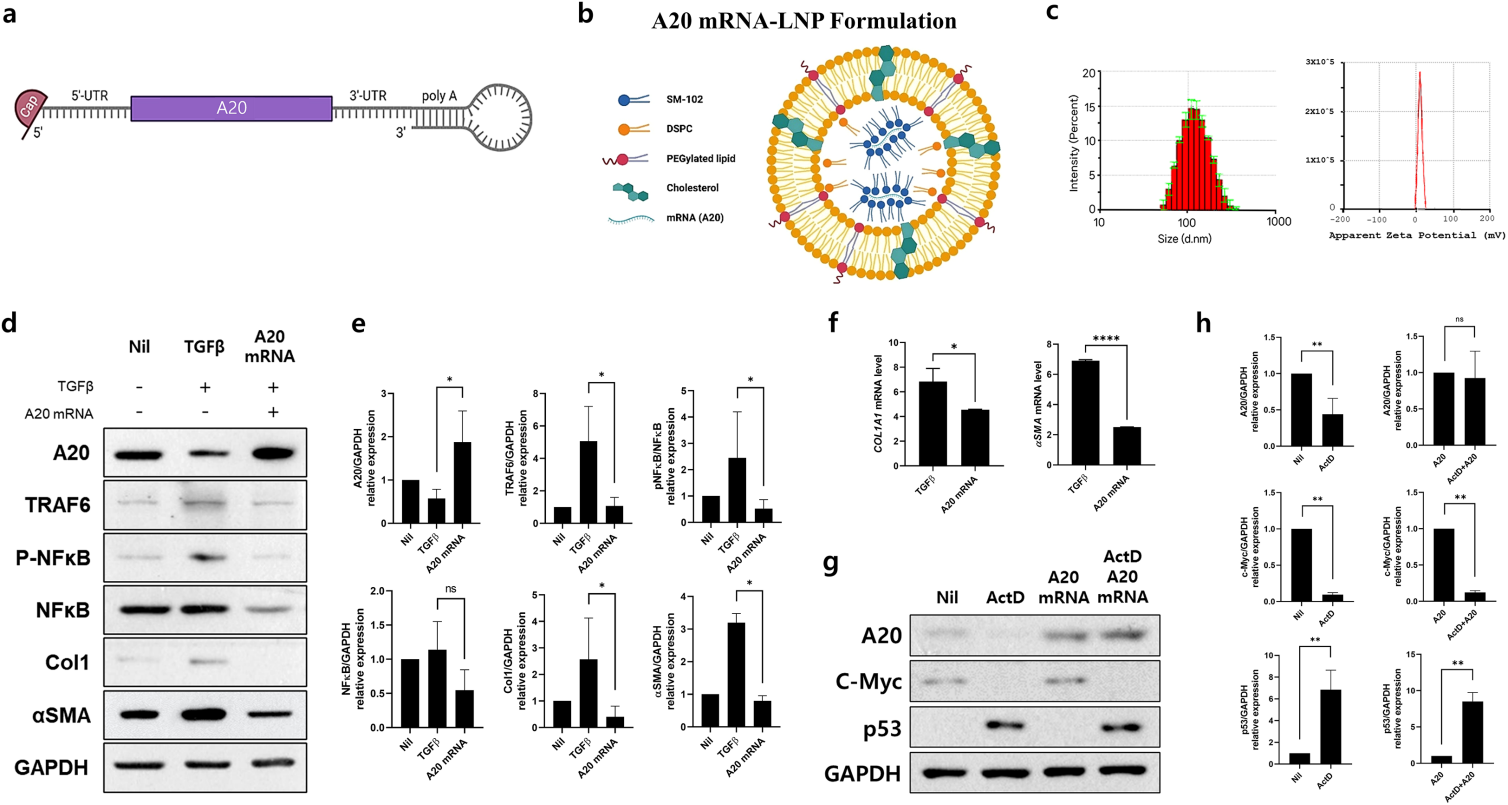
A20 mRNA-LNP Production and Expression *In Vitro*: Suppression of TRAF6-NF-κB Signaling and Fibrosis Markers in Skin Fibroblasts. **(A)** A20 mRNA was cloned into a linear mRNA expression construct, featuring a loop structure at the poly **(A)** tail. **(B)** A20 mRNA-LNP was formulated with four lipids, including the ionizable lipid SM-102. **(C)** Characterization of A20 mRNA-LNP, including particle size and zeta potential. Human dermal fibroblast (HDF) cells were transfected with either empty mRNA or A20 mRNA and serum-starved for 16–18 h **(D, E)** A20, TRAF6, pNF-κB, NF-κB, Col1, αSMA protein levels were measured in HDF cells treated with TGF-β (20 ng/mL) for 48 h GAPDH served as the loading control. Bar graphs show the pooled results of three independent experiments, with data presented as mean ± SD (n = 3). Statistical significance was determined by one-way ANOVA **(D, E)** (*p < 0.05; ns, not significant). **(F)** mRNA levels of *Col1A1* and *αSMA* in HDFs. Human dermal fibroblasts (HDFs) were transfected with the indicated A20 mRNAs and, stimulated with TGF-β (20 ng/mL) for 24 hours. Total RNA was extracted, and *Col1A1* and *αSMA* mRNA levels were analyzed by real-time PCR. **(G, H)** Protein expression of A20, p53, and c-Myc in Human dermal fibroblasts (HDFs). HDFs were treated with Actinomycin D (5 μg/mL) and subsequently transfected with the indicated A20 mRNAs. Four hours post-transfection, cells were stimulated for an additional 24 hours. Representative western blots are shown. Bar graphs summarize pooled results from five independent experiments and are presented as mean ± SD (n = 5). Statistical significance was determined by Mann-Whitney test **(F–H)** (****p < 0.0001; **p < 0.01; *p < 0.05; ns, not significant).

The A20 coding sequence was optimized using codon optimization services provided by GenScript (Piscataway, NJ, USA). The optimized sequence was synthesized by Cosmogenetech (Seoul, Korea) and subsequently cloned into the CUK3.1 mRNA expression platform (11). To generate A20 mRNA, the gene was inserted into a capped mRNA expression construct incorporating a loop structure at the poly(A) tail (**Figure 1A**). The DNA template was linearized using NotI (R001H, Enzynomics, Daejeon, Korea) and transcribed using the EZ T7 High Yield *In Vitro* Transcription Kit (EZ027S, Enzynomics) according to the manufacturer’s protocol. Capping was performed with SC101 (STPharm, Siheung, South Korea), and uridine triphosphate was substituted with N1-methyl-pseudouridine (N-1081, Trilink, San Diego, CA, USA) to enhance mRNA stability and translation. Double-stranded RNA contaminants were removed using a cellulose-containing column, as previously described (12).

### Cell culture and A20 mRNA transfection

Human dermal fibroblasts (HDFs) and human embryonic kidney 293 (HEK293) cells were cultured to 70–80% confluence in Dulbecco’s modified Eagle’s medium supplemented with 10% fetal bovine serum (#12800017 for HDFs and #16000044 for HEK293, Thermo Fisher Scientific, Waltham, MA, USA) and 100 U/mL penicillin–streptomycin (#15140-122, Gibco, Carlsbad, CA, USA). Cells were maintained in a humidified incubator at 37°C with 5% CO_2_. Transfections were performed using Lipofectamine™ MessengerMAX™ Transfection Reagent (LMRNA003, Thermo Fisher Scientific) according to the manufacturer’s instructions. For transfection, the culture medium was replaced with fresh Dulbecco’s modified Eagle’s medium containing 10% fetal bovine serum, and 2 µg of plasmid DNA was added. Transfected cells were then treated with recombinant human TGF-β (20 ng/mL; #100-21C-10, Thermo Fisher Scientific) for 48 h. For transcriptional inhibition experiments, cells were pretreated with actinomycin D (5 µg/mL; #A1410, Sigma-Aldrich, St. Louis, MO, USA) for 30 min prior to A20 mRNA-LNP transfection. ActD treatment was maintained during transfection, and cells were subsequently cultured for 24 h.

### LNP formulation and characterization

The lipid components were dissolved in ethanol at a molar ratio of 50:10:38.5:1.5, consisting of SM-102, distearoylphosphatidylcholine, cholesterol, and 1,2-dimyristoyl-rac-glycero-3-methoxypolyethylene glycol-2000. The mRNA was dissolved in a 50 mM sodium citrate buffer. Lipid nanoparticles (LNPs) were formulated using an Encell Master (Enparticle, Busan, South Korea) by mixing the aqueous and organic solutions at a 3:1 ratio. The resulting LNP solution was then concentrated via ultrafiltration using an Amicon ultracentrifugal filter (UFC9030, Merck Millipore, Billerica, MA, USA), following the manufacturer’s instructions. The size and zeta potential of the LNPs were assessed using a Zetasizer Nano ZS (Malvern Panalytical, Malvern, UK), with the mRNA diluted in water.

### Western blot

Antibodies targeting A20 (ab13597, Abcam, Cambridge, UK), TRAF6 (SC-8409, Santa Cruz Technologies, Santa Cruz, CA, USA), NF-κB (ab16502, Abcam), DREAM (SC-166916, Santa Cruz Technologies), SMAD2 (ab33875, Abcam), Col1a1 (PA5-29569, Invitrogen, CA, USA) αSMA (ab7817, Abcam), c-Myc (SC-40, Santa Cruz), p53 (SC-6243, Santa Cruz) and GAPDH (ab181602, Abcam) were used to measure the corresponding protein levels by Western blotting (SNAP i.d. Protein Detection System, SNAP2BHMB050, Merck Millipore). Protein concentrations were determined using the bicinchoninic acid assay (23235, Thermo Fisher Scientific). Samples were separated on 4–12% sodium dodecyl sulfate–polyacrylamide gels and transferred to nitrocellulose membranes (Amersham Pharmacia, Uppsala, Sweden). Band densities were quantified using image capture densitometry.

### Quantitative polymerase chain reaction

The mRNA expression levels of *COL1A1* (5’-CATAAAAGGCCCACTACCCAAC-3’/5’-ACCTTGCTCTCCTCTTACTGC-3’) and *αSMA* (5’-TGGGTGACGAAGCACAGAGC-3’/5’-CTTCAGGGGCAACACGAAGC-3’) were normalized to the expression of β-actin. Total mRNA was extracted using TRIzol (Molecular Research Center, Cincinnati, OH, USA). cDNA was synthesized using a reverse transcription system (TaKaRa, Shiga, Japan). qPCR was performed using LightCycler FastStart DNA Master SYBR Green I (TaKaRa), following the manufacturer’s instructions.

### Bleomycin-induced SSc

Seven-week-old male SKG mice were purchased from Orient Bio Inc. (Seongnam, Korea) and housed under specific-pathogen-free conditions at the Institute of Medical Sciences, Catholic University of Korea, with ad libitum access to water and standard mouse chow (Ralston Purina, St. Louis, MO, USA). To induce SSc, bleomycin (HY-17565A, MedChemExpress, USA) was dissolved in phosphate-buffered saline and administered subcutaneously at a dose of 50 µg in 100 µL daily for 3 weeks. All *in vivo* studies were conducted in three independent experiments, each comprising five mice per group. Animal experiments were performed in accordance with the Laboratory Animal Welfare Act, the Guide for the Care and Use of Laboratory Animals, and the Guidelines and Policies for Rodent Experiments of the Institutional Animal Care and Use Committee of the Catholic University of Korea.

### A20 mRNA-LNP treatment in SSc mice

A20 mRNA-LNP was administered via intramuscular injection beginning 3 days after the initiation of bleomycin treatment. The treatment regimen involved weekly injections of 10 µg of A20 mRNA-LNP for 6 weeks. The control group received LNP alone, following the same dosing schedule and route of administration.

### Flow cytometry

Th2 and Th17 cells were stained with PC5.5-CD4 (45-0042-82, eBioscience, San Diego, CA, USA), PE-IL-4 (554435, eBioscience), and APC-IL17 (17-7177-81, eBioscience). Spleen cells were stained with antibodies against CD4, IL-4, and IL-17 to identify Th2 and Th17 cell populations, and analyzed using a FACSCalibur flow cytometer (BD Biosciences, San Jose, CA, USA).

### Histopathological analysis

After euthanasia, skin and lung tissues were collected, fixed in 10% formalin (#HT501320, Sigma), and embedded in paraffin. Sections (5 µm) were prepared using a microtome (Leica Biosystems) and stained with hematoxylin and eosin (H&E), Masson’s trichrome, and Sirius Red. Dermal thickness was defined as the distance between the epidermal–dermal and dermal–adipose junctions. The severity of lung fibrosis was semi-quantitatively assessed, as described by Ashcroft et al. (13). Briefly, lung fibrosis was scored on a scale of 0–8: grade 0, normal lung; grade 1, minimal fibrous thickening of alveolar or bronchiolar walls; grade 3, moderate thickening without structural damage; grade 5, definite fibrosis with fibrous bands or small masses; grade 7, severe distortion with large fibrotic areas; and grade 8, complete fibrous obliteration. Grades 2, 4, and 6 represented intermediate changes. Fibrotic areas in Masson’s trichrome- and Sirius Red–stained sections were quantified using ImageJ. In skin tissues, the collagen-positive area was expressed as the percentage of blue (Masson) or red (Sirius Red) area within the dermis. In lung tissues, the collagen-positive area was calculated in the same manner and expressed as the percentage of fibrotic tissue per field. All histological analyses were performed under blinded conditions.

### Hydroxyproline assay

For determination of Skin dermal and Lung lobes collagen content, colorimetric quantification of hydroxyproline was performed in a small skin and lung biopsy (5 mm diameter) taken from every animal. Briefly, frozen skin tissues were dehydrated, weighed, and hydrolyzed in 12 N HCl at 120°C for 3 h (BM-HYP-100, Biomax). After hydrolyed, the samples were mixed with chloramine T reagent and incubated for 5 min at room temperature. Next, a reagent mixed with perchloric acid/isopropanol solution and 2X DMAB was added, and the sample was incubated at 60°C for an additional 90 minutes. The absorbance was measured at 560 nm in duplicate with a microplate spectrophotometer.

### Immunofluorescence staining

The skin and lung tissues from the mice undergoing immunofluorescence analysis were embedded in paraffin and then sliced into 5 μm sections. After blocking with 10% NGS (Normal goat serum with PBS), the sections were placed in a humid chamber and incubated with the corresponding primary antibodies overnight at 4°C. The following primary antibodies were used: anti-CD4 (14-9766-82, Invitrogen), anti-IL-4 (PA5-25165, Invitrogen), and anti-IL-17 (PA5-114455, Invitrogen). Subsequently, the sections were incubated with fluorescence-labeled secondary antibodies (Mouse anti-rat FITC, Mouse anti-rabbit APC) at room temperature in the dark for 2 hours. Finally, the sections were stained with DAPI solution (Servicebio G1012) for 30 min to visualize the cell nuclei. After quenching the autofluorescence of the tissue, the sections were mounted with anti-fade mounting medium for fluorescence (CS70330-2, Dako). Fluorescence images were captured using an Confocal Laser Scanning Microscope(LSM700, ZEISS), and at least three representative fields were selected for analysis using Image J software.

### Immunohistochemistry

Paraffin-embedded tissue sections were incubated at 4°C with the following primary monoclonal antibodies: anti-A20 (ab13597, Abcam), anti-TRAF6 (SC-8409, Santa Cruz Technologies), anti-NF-κB (3033S, Cell Signaling), anti-DREAM (SC-166916, Santa Cruz Technologies), anti-SMAD2 (ab33875, Abcam), anti-TGF-β (BS-0086R; Fisher Scientific), anti-procollagen (ab64409, Abcam), and anti-αSMA (ab7817, Abcam). The sections were then incubated with biotinylated secondary antibodies and subsequently with streptavidin–peroxidase complex for 30 min. Reaction products were developed using 3,3-diaminobenzidine chromogen (K3468, Dako Corp., Carpinteria, CA, USA).

### Statistical analysis

Results are presented as means ± standard error of mean (SEM). Data were analyzed using Student’s t-test, the Mann–Whitney U test, or one-way ANOVA, performed with Prism 8 software (GraphPad Software Inc., San Diego, CA, USA). P values < 0.05 (two-tailed) were considered statistically significant.

## Results

### *In vitro* synthesis and delivery of A20 mRNA-LNPs: inhibiting TRAF6/NF-κB signaling and fibrosis in HDFs

To generate A20 mRNA, the A20 gene was inserted into a capped mRNA expression construct containing a loop structure at the poly(A) tail (**Figure 1A**). The A20 mRNA was encapsulated in LNPs composed of four lipid components, as described in the Methods section (**Figure 1B**). The resulting A20 mRNA-LNPs displayed a Z-average particle size of approximately 110.88 ± 0.7 nm and a zeta potential of 10.4 ± 0.523 (**Figure 1C**). These results demonstrate the successful development of an A20 mRNA-LNP formulation with stable physicochemical properties.

To evaluate the inhibitory effect of A20 mRNA on TRAF6/NF-κB signaling-mediated fibrosis, HDFs were transfected with A20 mRNA to induce A20 expression. The cells were then stimulated with TGF-β to activate TRAF6/NF-κB signaling and induce a fibrotic response. Western blot analysis showed that A20 mRNA transfection increased A20 protein levels while reducing the expression of TRAF6, pNF-κB, αSMA, and Col1 (**Figures 1D, E**). Consistently, qPCR analysis in HDFs demonstrated that A20 mRNA transfection significantly reduced TGF-β (20 ng/mL)-induced upregulation of *COL1A1* and *αSMA*, 24 hours after stimulation (**Figure 1F**). These data confirm that A20 overexpression suppresses fibrotic gene transcription downstream of TGF-β signaling, complementing the observed inhibition of pro-fibrotic proteins. Together, these findings demonstrate that A20 effectively inhibits both upstream TGF-β/NF-κB signaling and downstream fibrotic gene expression, highlighting its potential as a negative regulator of fibroblast activation. To evaluate whether A20 delivery restores protein expression independently of host transcription, fibroblasts were pretreated with actinomycin D (ActD, 5 µg/mL) prior to A20 mRNA-LNP administration. ActD treatment markedly reduced endogenous A20 and c-Myc protein levels, while inducing p53 accumulation, consistent with its role as a marker of transcriptional stress (**Figure 1G**). Notably, subsequent A20 mRNA-LNP delivery significantly restored A20 expression despite transcriptional inhibition. These findings demonstrate that exogenous A20 mRNA-LNP enables protein restoration under transcriptionally repressive conditions (**Figure 1H**).

### A20 mRNA-LNP suppresses fibrosis in SSc

**Figure 2:**
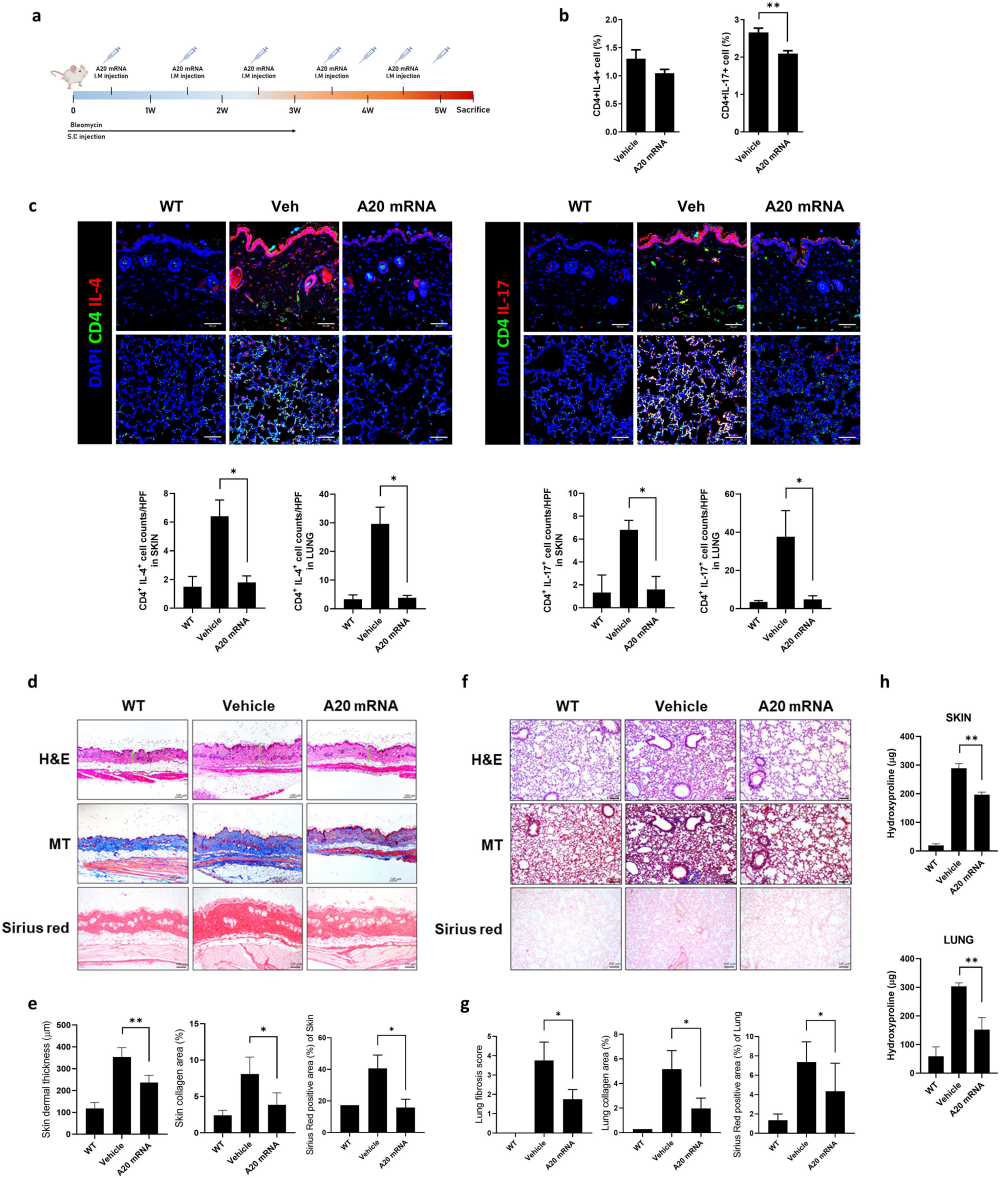
A20 mRNA Suppressed Immune Cell Activation and Skin and Lung Fibrosis in a Mouse Model of Systemic Sclerosis (SSc). **(A)** A murine model of SSc was generated as described in the schematic. Mice were injected subcutaneously (S.C.) with bleomycin daily for 3 weeks to induce fibrosis. Mice received a total of seven intramuscular (I.M.) injections of A20 mRNA-LNP (n = 5) or vehicle control (n = 5), administered once or twice weekly over 6 weeks. All mice were sacrificed at week 6 for subsequent analyses. **(B)** At the 6-week sacrifice time point, the frequency of Th2 (IL-4+ CD4+) and Th17 (IL-17+ CD4+) cells in the spleen was analyzed by flow cytometry. **(C)** Immunofluorescence analysis of CD4^+^ T cell subsets in skin and lung tissues. Tissue sections from WT mice, bleomycin-induced fibrotic mice treated with vehicle, and bleomycin-induced fibrotic mice treated with A20 mRNA-LNP were stained for CD4 (green), IL-4 or IL-17 (red), and nuclei (DAPI, blue). Representative confocal images are shown (scale bar, 50 μm). Quantification of CD4^+^IL-4^+^ and CD4^+^IL-17^+^ cells per high-power field (HPF) is shown in the adjacent bar graphs. Original magnification: 200×; scale bar: 50 μm. **(D, E)** Representative skin sections stained with hematoxylin and eosin (H&E) showing dermal thickening, Masson’s Trichrome (MT) demonstrating collagen deposition, and Sirius Red highlighting fibrotic area. Dermal thickness and percentage of fibrotic area were quantified. **(F, G)** Representative lung sections stained with H&E showing fibrotic morphology and alveolar wall thickening, MT demonstrating collagen deposition, and Sirius Red highlighting fibrotic area. Fibrotic area was quantified accordingly. Original magnification: 200×; scale bar: 100 μm. **(H)** Hydroxyproline levels were measured in skin (top) and lung (bottom) tissues from WT mice, bleomycin-induced fibrotic mice treated with vehicle, and bleomycin-induced fibrotic mice treated with A20 mRNA-LNP. For quantification, three fields per mouse were analyzed (n = 5 mice per group). Data are presented as mean ± SD from one representative experiment of three independent experiments. The statistical significance of all graphs was determined by one-way ANOVA **(B–H)**. (*p < 0.01; p < 0.05; ns, not significant).

The anti-fibrotic effects of A20 mRNA-LNP were evaluated to assess its therapeutic potential in an SSc mouse model. SSc was induced by bleomycin, which was administered daily for 3 weeks, and A20 mRNA-LNP was given over seven doses during a 6-week period (**Figure 2A**). Treatment with A20 mRNA-LNP resulted in a reduction in the activity of Th2 (Vehicle: 3.49%, A20 mRNA: 2.94%) and Th17 cells (Vehicle: 3.81%, A20 mRNA: 2.73%), which are known to promote fibrotic responses in SSc (**Figure 2B**). Although a decreasing trend in Th2 cells was observed, this difference did not reach statistical significance. In bleomycin-induced fibrotic mice, A20 mRNA-LNP treatment significantly reduced CD4^+^IL-4^+^ and CD4^+^IL-17^+^ T cell infiltration in the skin and lung compared to vehicle, indicating suppression of Th2- and Th17-mediated responses (**Figure 2C**). Histological analyses were performed to assess fibrosis in the SSc model. In skin sections, H&E staining demonstrated dermal thickening, Masson’s Trichrome (MT) staining revealed collagen deposition, and Sirius Red staining quantified the fibrotic area (**Figures 2D, E**). In lung sections, H&E staining showed fibrotic changes with alveolar wall thickening, MT highlighted collagen accumulation, and Sirius Red staining measured the extent of fibrosis (**Figures 2F, G**). Hydroxyproline levels were measured in skin and lung tissues to assess collagen deposition (**Figure 2H**). Administration of A20 mRNA-LNP significantly decreased hydroxyproline levels in both skin and lung, demonstrating its anti-fibrotic effect. Collectively, these findings indicate that A20 mRNA-LNP effectively suppresses fibrosis in the SSc model.

### A20 mRNA-LNP inhibits TRAF6/NF-κB signaling-mediated skin and lung fibrosis

**Figure 3:**
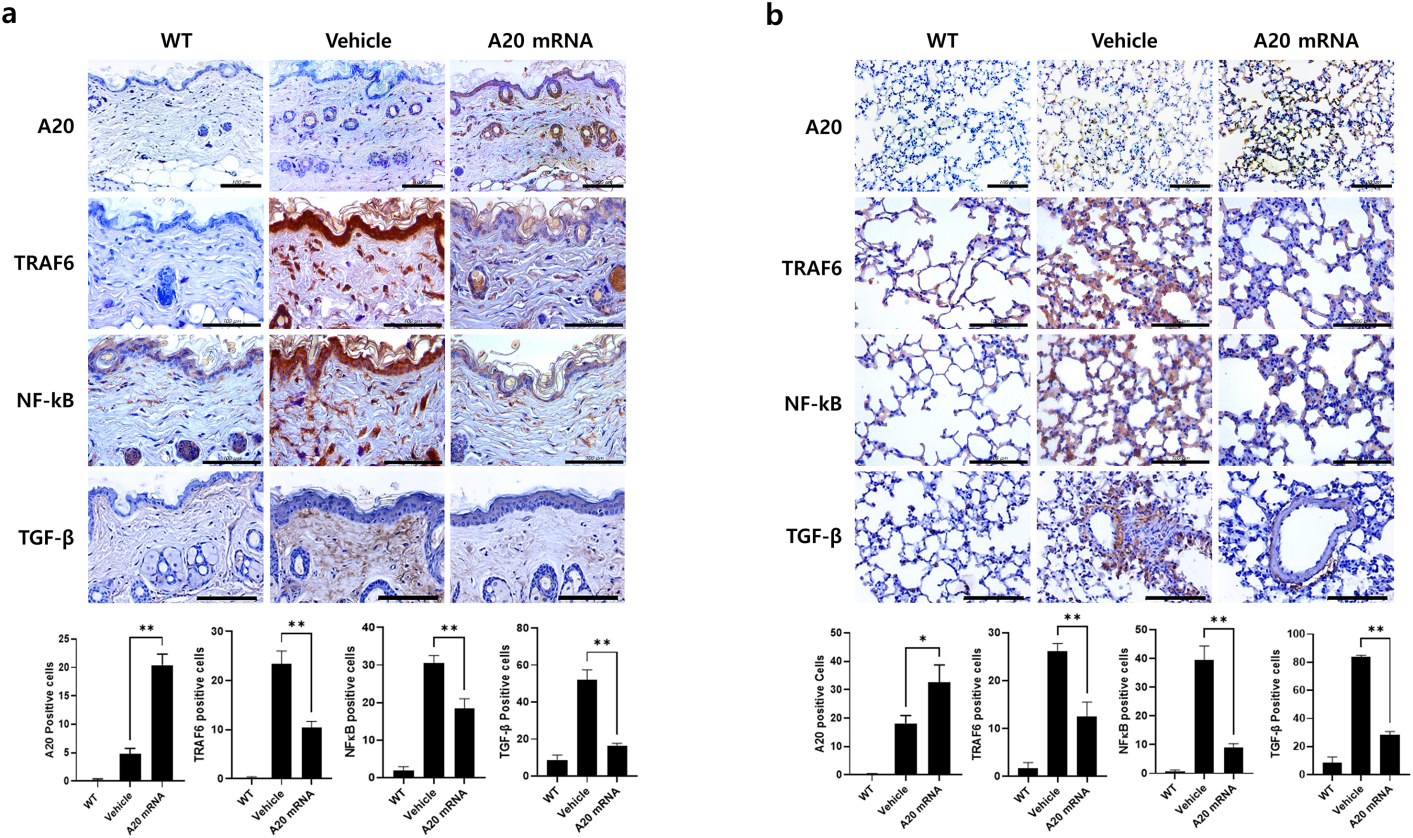
A20 mRNA Therapy Inhibited TRAF6/NF-κB Signaling and TGF-β Cytokine Expression in Skin and Lung Tissues from Systemic Sclerosis. Skin and lung tissues collected at the 6-week sacrifice time point were analyzed. **(A)** Skin sections were immunohistochemically stained for A20, TRAF6, NF-κB, and TGF-β. **(B)** Lung sections were stained in parallel with the same antibodies to evaluate A20 expression and fibrotic signaling molecules. For quantification, three fields per mouse were analyzed (n = 5 mice per group), and the number of antibody-positive cells was presented as mean ± SD. Original magnification: 400×; scale bar: 100 μm. Bar graphs represent data from one of three independent experiments, with statistical significance determined by one-way ANOVA (*P < 0.05, **P < 0.01; **(A, B)**.

To investigate the inhibitory effect of A20 overexpression on TRAF6/NF-κB signaling-mediated fibrosis, immunohistochemistry analyses were performed in both skin and lung tissues. In skin sections, administration of A20 mRNA-LNP increased A20 expression, accompanied by a relative decrease in TRAF6 and NF-κB levels compared to the vehicle group (**Figure 3A**). Expression of the fibrotic factor TGF-β was also reduced in A20-overexpressing skin. In lung sections, A20 mRNA-LNP treatment similarly enhanced A20 expression and reduced the levels of TRAF6, NF-κB, and TGF-β compared with the vehicle group (**Figure 3B**). These findings demonstrate that A20 overexpression via A20 mRNA-LNP suppresses the TRAF6/NF-κB pathway in both skin and lung tissues in the SSc model.

### A20 mRNA-LNP modulates the DREAM–SMAD2 pathway to attenuate fibrosis in SSc

**Figure 4:**
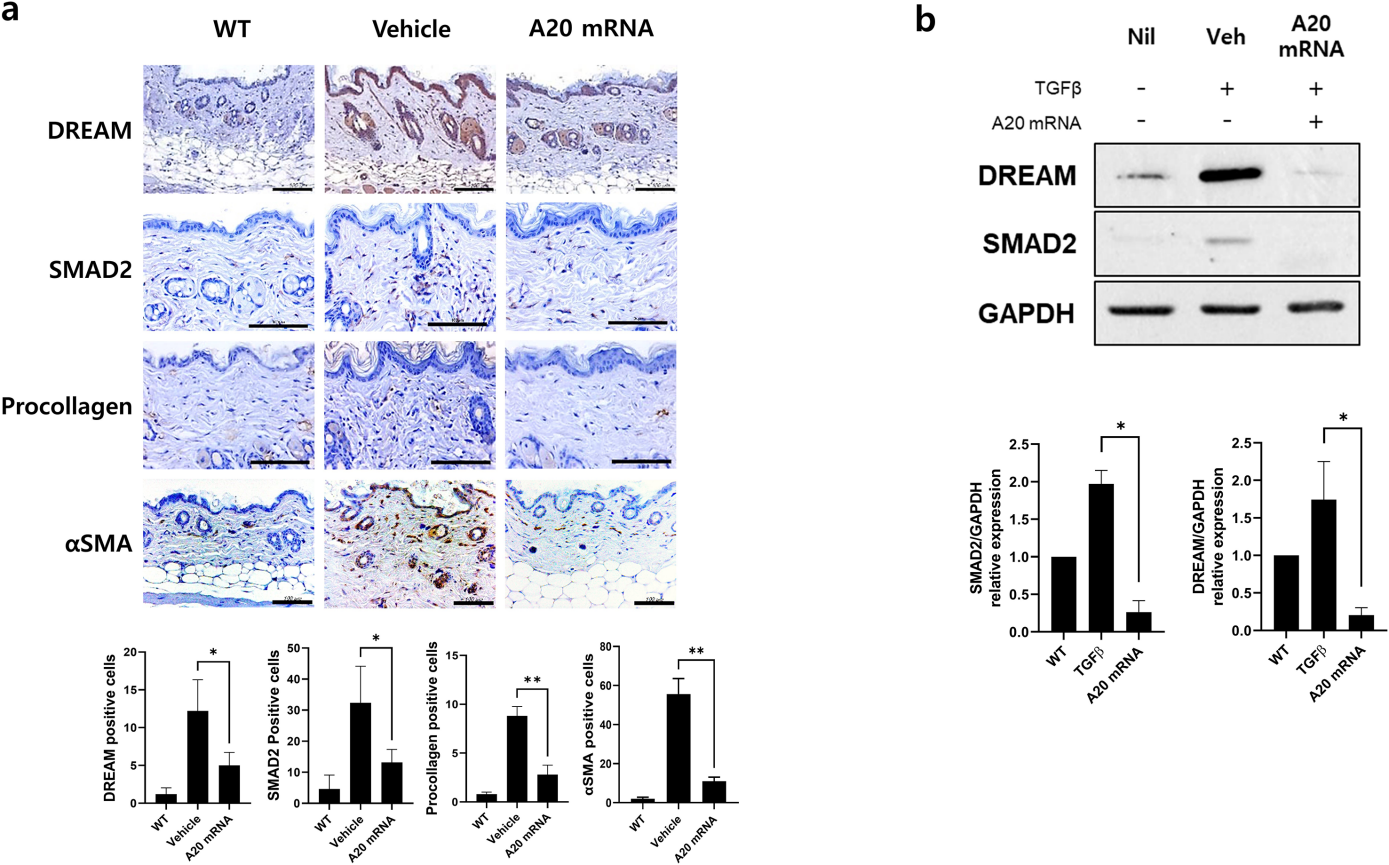
A20 mRNA Therapy Suppressed the Expression of DREAM, a Known A20 Inhibitory Molecule, and Reduced SMAD2, αSMA, and Procollagen in Systemic Sclerosis Skin Tissue and Human Skin Fibroblasts. **(A)** Skin and lung sections were stained with antibodies against DREAM, SMAD2, procollagen, and αSMA. The number of antibody-positive cells (mean ± SEM) is plotted. Original magnification: 400×; scale bar: 100 μm. **(B)** The protein levels of DREAM and SMAD2 were measured in cells treated with TGF-β (20 ng/mL) for 48 h GAPDH served as the validation control. For quantification, three fields per mouse were analyzed (n = 5 mice per group). Data are presented as mean ± SD from a representative experiment of three independent experiments. Statistical significance was determined by one-way ANOVA **(A, B)**. (*P < 0.05; **P < 0.01).

Recent studies have suggested an inverse relationship between A20 and the DREAM complex, with the latter promoting fibrosis by suppressing A20 expression. This study investigated the hypothesis that A20 mRNA-LNP regulates the DREAM–SMAD2 pathway in SSc. In the SSc mouse model, the vehicle group exhibited increased DREAM expression in skin tissues compared to wild-type controls, which is consistent with observations in SSc patients. Treatment with A20 mRNA-LNP effectively suppressed bleomycin-induced DREAM upregulation (**Figure 4A**). Inhibition of DREAM also suppressed SMAD2, a downstream target of DREAM, and SMAD2-mediated fibrotic factors, such as Procollagen and α-SMA. To further validate this mechanism, the effect of A20 overexpression in HDFs was examined. The results demonstrated significant inhibition of DREAM–SMAD2 activity in A20-overexpressing HDFs (**Figure 4B**), confirming the *in vivo* findings.

### A20 mRNA-LNP suppresses lung fibrosis in SSc

**Figure 5:**
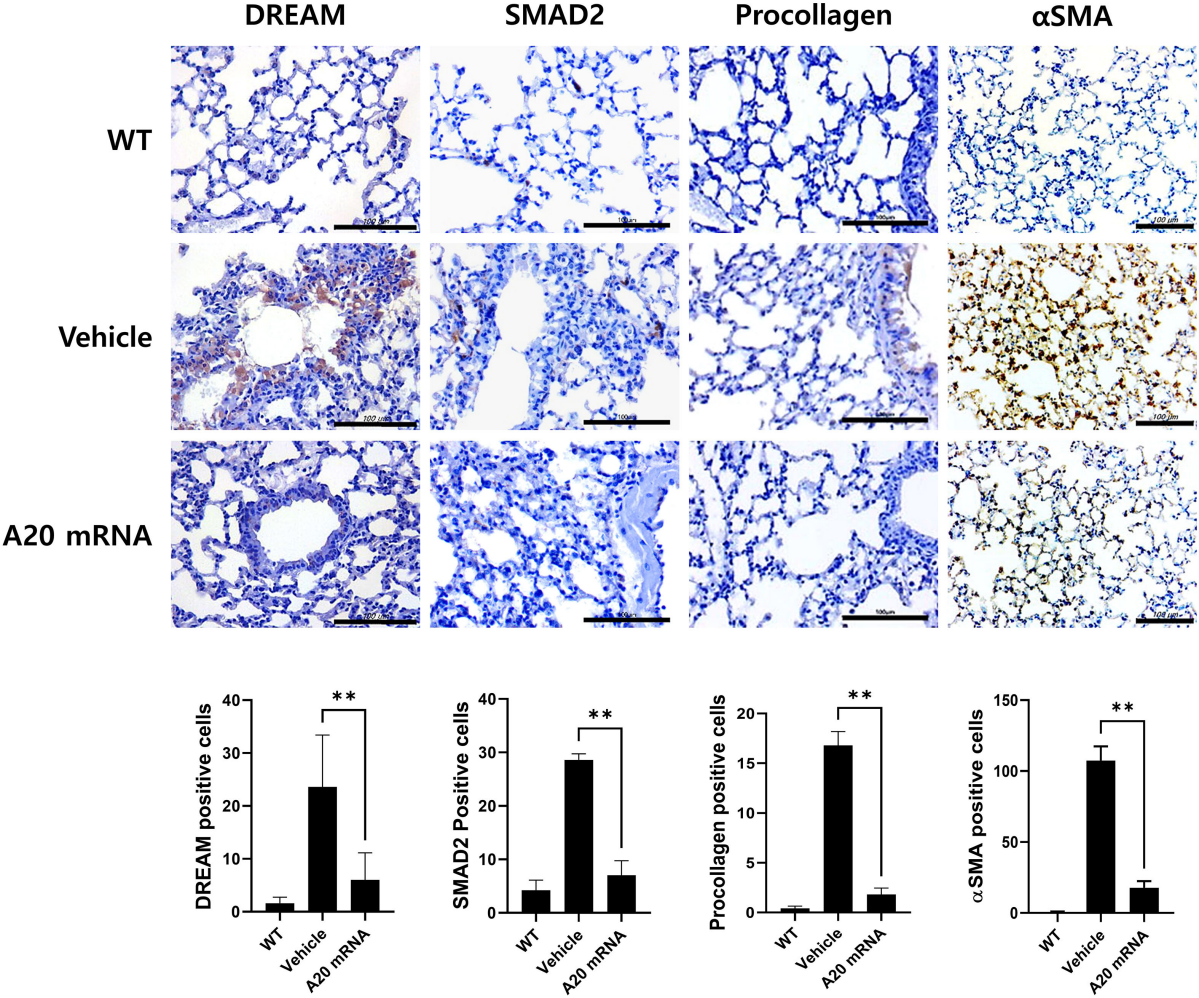
A20 mRNA Therapy Reduces Fibrosis in Systemic Sclerosis Lung Tissue by Inhibiting DREAM Expression. Lung sections were stained with antibodies against DREAM, SMAD2, procollagen, and αSMA. For quantification, three fields per mouse were analyzed (n = 5 mice per group), and the number of antibody-positive cells is plotted as mean ± SEM. Original magnification: 400×; scale bar: 100 μm. Bar graph shows data from one representative experiment (**P < 0.01). The statistical significance of all graphs was determined by one-way ANOVA **(A)**. The English in this document has been checked by at least two professional editors, both native speakers of English. For a certificate, please see: http://www.textcheck.com/certificate/bAoEK8.

In the SSc mouse models, the vehicle group exhibited increased DREAM expression in lung tissue compared to wild-type controls, consistent with the pattern observed in skin tissue. In the A20 mRNA-LNP treatment group, lung fibrosis was effectively inhibited by modulating the DREAM–SMAD2 pathway in the bleomycin-induced SSc model (**Figure 5**). These results suggest that A20 mRNA-LNP not only inhibits fibrosis mechanisms in skin tissues but also in lung tissues, with the regulation of the DREAM–SMAD2 pathway playing a crucial role in this process.

### Proposed mechanism by which A20 mRNA therapy attenuates fibrosis in systemic sclerosis

**Figure 6:**
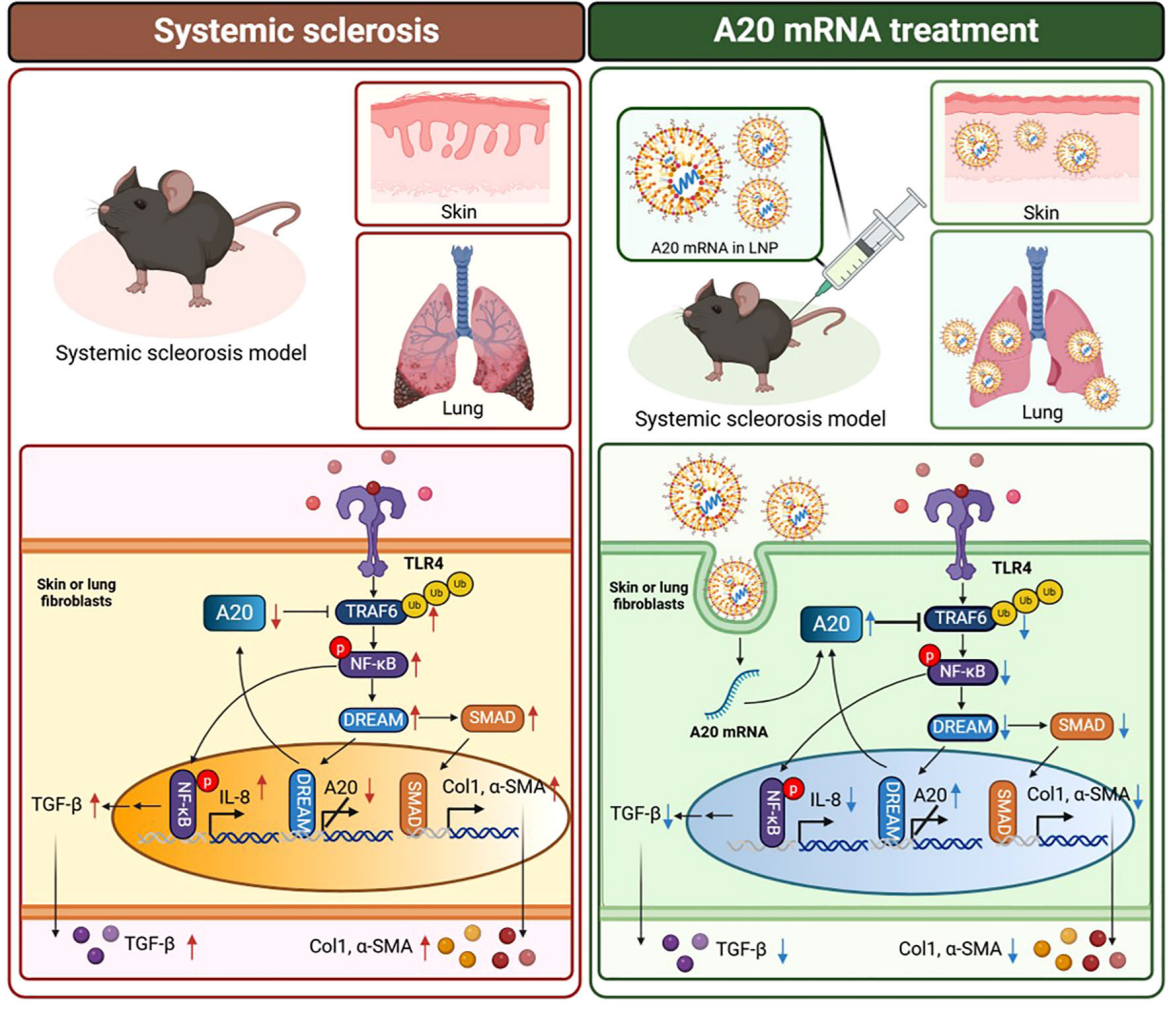
Proposed mechanism by which A20 mRNA therapy attenuates fibrosis in systemic sclerosis. In the systemic sclerosis condition (left), activation of TLR4 initiates TRAF6-mediated NF-κB signaling and upregulates TGF-β, IL-8, collagen type I (Col1), and α-smooth muscle actin (α-SMA), thereby promoting fibroblast activation and extracellular matrix (ECM) accumulation. In contrast, A20 mRNA encapsulated in lipid nanoparticles (LNPs) (right) restores A20 protein expression, leading to inhibition of TRAF6-dependent NF-κB phosphorylation, suppression of DREAM and SMAD pathways, and subsequent reduction of fibrotic markers in skin and lung fibroblasts. Downward arrows indicate decreased expression following A20 mRNA treatment. The schematic was created with BioRender.com.

To provide a conceptual overview of the therapeutic mechanism, we illustrated a schematic comparison between the fibrotic signaling pathways observed in systemic sclerosis and those modulated by A20 mRNA treatment (**Figure 6**). In the systemic sclerosis condition, activation of TLR4–TRAF6–NF-κB signaling and subsequent induction of TGF-β promote fibroblast activation and extracellular matrix (ECM) deposition, leading to excessive collagen and α-SMA expression. In contrast, delivery of A20 mRNA via lipid nanoparticles restored A20 protein expression in fibroblasts, suppressing TRAF6-dependent NF-κB activation and downregulating TGF-β/SMAD and DREAM signaling. These changes collectively attenuated pro-fibrotic gene expression and ameliorated skin and lung fibrosis in the systemic sclerosis model.

## Discussion

Fibrosis is a hallmark of systemic sclerosis (SSc), and our study demonstrates that A20 mRNA therapy effectively modulates key fibrotic pathways.

A20, a ubiquitin-editing enzyme encoded by TNFAIP3, inhibits TRAF6-dependent NF-κB signaling, thereby regulating fibroblast activation, collagen and α-smooth muscle actin (α-SMA) production, and extracellular matrix deposition (14, 15). In SSc fibroblasts, A20 expression is suppressed primarily by the transcriptional repressor DREAM (8).

Previous approaches to upregulate A20, such as AdipoRon, rely on intact endogenous transcription and suggest limited effectiveness in SSc, particularly in A20-deficient fibroblasts (9). By contrast, A20 mRNA therapy bypasses transcriptional regulation, directly restoring functional protein and inhibiting downstream fibrotic signaling. Our ActD transcription inhibition experiments further confirmed that A20 mRNA delivery restored protein expression even under transcriptionally repressive conditions, providing direct evidence for transcription-independent activity. These findings highlight a key advantage of mRNA-based therapeutics, in which functional protein production can be achieved irrespective of impaired or dysregulated host transcriptional machinery.

In addition to this transcription-independent restoration, our study revealed that A20 mRNA therapy downregulates DREAM, a transcriptional repressor of A20. The combination of restoring A20 and suppressing DREAM provides a dual mechanism of action that offers a more targeted intervention than strategies acting on single downstream effectors. Our findings indicate that A20 mRNA therapy not only restores A20 levels but also inhibits TRAF6/NF-κB signaling, reduces TGF-β expression, and downregulates DREAM, suggesting a previously unrecognized feedback mechanism in fibrosis regulation. Our results align with prior studies showing that A20 deletion exacerbates TGF-β-induced fibrosis, while A20 overexpression alleviates these effects, and that DREAM knockdown enhances A20 expression and mitigates fibrotic responses (8). The inverse correlation between A20 and DREAM observed in our study suggests a regulatory feedback loop, highlighting the therapeutic potential of simultaneously modulating both molecules in SSc and other fibrotic diseases.

TNFAIP3 polymorphisms have been associated with SSc and linked to reduced A20 expression, thereby contributing to NF-κB hyperactivation and fibrosis (9). Recent studies further demonstrate that A20 expression is downregulated in skin and lung tissues of SSc patients, as well as in fibroblasts, correlating with increased expression of profibrotic mediators such as Col1a1 and fibronectin (9). Functionally, A20 inhibits TGF-β–induced Smad signaling and collagen/α-SMA expression in fibroblasts, underscoring its role as a critical anti-fibrotic regulator.

Although our study did not directly assess the functional consequences of specific SNPs, these findings collectively indicate that both genetic variants and the fibrotic microenvironment contribute to reduced A20 activity in SSc. To overcome such variability, we employed A20 mRNA therapy to restore protein expression. In the bleomycin-induced SSc model, A20 mRNA delivery effectively increased A20 levels, suppressed profibrotic cytokines, and attenuated fibrosis.

Collectively, these findings suggest that future studies utilizing patient-derived cells harboring specific TNFAIP3 polymorphisms would be valuable to determine whether A20 mRNA therapy can compensate for SNP-associated A20 insufficiency in fibrotic disease. The transient and controllable nature of mRNA therapeutics offers advantages over gene therapy by minimizing permanent genomic alterations while providing potent modulation of disease pathways (16). A20 mRNA therapy is compatible with this approach, allowing selective inhibition of pathological NF-κB activation without broadly affecting physiological processes.

Moreover, targeting TRAF6/NF-κB upstream of TGF-β provides a more selective intervention than conventional anti-fibrotic treatments, which often act downstream and can induce off-target systemic effects (17). Potential combination strategies further support the translational relevance of A20 mRNA therapy. Its distinct mechanism allows pairing with existing anti-fibrotic agents, such as pirfenidone or nintedanib, to enhance therapeutic efficacy and expand treatment options. By simultaneously modulating immune-driven inflammation and fibroblast dysfunction, A20 mRNA therapy offers a strategy with distinct mechanistic advantages for fibrosis treatment. The subcutaneous bleomycin model reliably induces skin fibrosis and, although lung involvement is less pronounced, our study also observed mild pulmonary fibrosis, highlighting both its utility and its limitations. Safety concerns related to NF-κB inhibition must also be evaluated to ensure that normal immune responses are preserved while pathological inflammation is suppressed (18).

Additionally, the precise molecular mechanism by which A20 downregulates DREAM remains to be elucidated, including potential interactions with other proteins or epigenetic regulators. Optimizing delivery strategies is critical for clinical translation. While LNP-based delivery was effective in our study, improving tissue-specific targeting, minimizing off-target effects, and exploring inhalable formulations could enhance pulmonary transfection and therapeutic outcomes.

A promising direction for future research is the integration of new delivery platforms that enhance pulmonary targeting. Recent advancements in nanoparticle formulations, including surface modifications and inhalable delivery systems, have shown improved mRNA transfection in the lung epithelium, particularly with ionizable lipids and polymer-based approaches (19, 20). Combining these emerging delivery strategies with mRNA constructs encoding antifibrotic agents could lead to more effective and sustained treatments. Future studies should focus on optimizing these approaches to expand therapeutic options for pulmonary fibrosis in SSc.

## Conclusion

Our study demonstrates that A20 mRNA therapy effectively suppresses fibrosis by restoring A20 expression, inhibiting TRAF6/NF-κB signaling, and downregulating DREAM in an *in vivo* model of SSc. This dual mechanism underscores A20 and DREAM as key regulatory nodes in fibrosis progression, suggesting that targeting both factors could offer a more effective therapeutic strategy than previously explored approaches. With the growing interest in mRNA-based therapies, our findings pave the way for further clinical development of A20 mRNA therapy as a novel intervention for SSc and other fibrotic diseases.

## Data Availability

The original contributions presented in the study are included in the article/supplementary files. Further inquiries can be directed to the corresponding author.
